# Bioinspired antireflective flexible films with optimized mechanical resistance fabricated by roll to roll thermal nanoimprint

**DOI:** 10.1038/s41598-021-81560-6

**Published:** 2021-01-28

**Authors:** Alejandra Jacobo-Martín, Mario Rueda, Jaime J. Hernández, Iván Navarro-Baena, Miguel A. Monclús, Jon M. Molina-Aldareguia, Isabel Rodríguez

**Affiliations:** 1 Madrid Institute for Advanced Studies in Nanoscience (IMDEA Nanoscience), Ciudad Universitaria de Cantoblanco, C/ Faraday 9, 28049 Madrid, Spain; 2 Madrid Institute for Advanced Studies in Materials (IMDEA Materials), C/ Eric Kandel 2, Tecnogetafe, Getafe, 28906 Madrid, Spain; 3 Department of Materials Science, Universidad Politécnica de Madrid, E. T. S. de Ingenieros de Caminos, 28040 Madrid, Spain

**Keywords:** Polymers, Surface patterning, Materials science, Nanoscience and technology

## Abstract

This work describes the fabrication process of moth eye antireflective poly (methyl methacrylate) transparent films via roll to roll thermal nanoimprint lithography. The process parameters are investigated and adjusted in order to obtain from a single moth-eye structured mold, a range of antireflective topographies that gradually vary their geometry from protruding to intruding nanocones. A correlation between the process parameters with the optical and mechanical properties of the films is established to illustrate the influence of the processing parameters and serve as guideline to produce antireflective flexible films with balanced properties and optimized performance adequate to the application environment. A finite element model is described predicting the mechanical behavior of the moth-eye PMMA imprinted nanostructures.

## Introduction

Energy harvesting from light as a renewable source is becoming increasingly important as our energy demands steadily increase at the same time that we need to move onto low-carbon sources to fight climate change. Towards this end, flexible photovoltaic (PV) devices have attracted enormous attention, particularly to power up all of the many portable electronic devices and other low energy devices we use in our daily life.

For these applications, flexible PVs would be more advantageous than the more established silicon base solar cells because today PV devices have become very efficient and can be made at a fraction of the cost. Furthermore, they are lightweight and can be made of various shapes and sizes and being flexible, they can be even fold up. Hence, they can be easily transported as portable devices or deployable for installation in remote places^1^.

For this type of organic flexible PV devices, there is a need to have efficient solutions for low-cost antireflective (AR) covers.

Subwavelength surface-relief structures like those inspired on the nanoscopic corneal nipples found in the moth eye and other insects^2,3^ have proven to be an efficient and low cost means to reduce light loses due to reflection and to improve light transmission.

These surfaces typically include arrays of hexagonally packed subwavelength nanocones that effectively create a gradual refractive index transition between the air and substrate. Incident light at this interface is gradually refracted inwards and consequently reflection is reduced while light transmittance is increased. Furthermore, these bioinspired moth-eye nanocone textures eliminate Fresnel reflections over a large spectral bandwidth and over omnidirectional angles of incidence^4,5^.

Several approaches have been employed to fabricate antireflective textures on polymeric films such as plasma etching^6^, replication techniques such as casting^7^, UV nanoimprint lithography (UV-NIL)^8^ or hot embossing technology, where the antireflective pattern is transferred from a rigid mold, generally silicon, to a thermoplastic film by thermal NIL (T-NIL)^9–12^.

Concerning large scale production of antireflective textures, roll to roll (R2R) nanoimprint lithography is a well suited technology due to its high throughput and nano scale resolution^13^. Thermal R2R-NIL has been applied for the fabrication of functional nano and micro structures on flexible films, based on thermoplastic polymers such as polycarbonate, cellulose acetate or PMMA^14–16^. Nonetheless, up to date, thermal R2R-NIL has not been employed to produce moth-eye AR surfaces on thermoplastic films. Today, the large scale fabrication of nanoimprinted antireflective films is performed by UV R2R or roll to plate NIL^17,18^. In UV R2R-NIL, the pattern is transferred onto a photoresist layer, previously coated generally on a PET web carrier, which is hardened upon crosslinking under UV illumination. Compared to R2R UV-NIL, the T-NIL approach presents some important advantages. Firstly, it is the lower cost of thermoplastics films compared to UV photoresins. It can be employed to pattern self-standing films directly on the material of choice, avoiding reflection losses occurring at the interface between the web carrier and photoresins^19^ and utilization of adhesion promoters^20^. Also, the utilization of a unique material with functional nanostructured surface eliminate problems related to volume contraction upon resin UV curing and differences in thermal expansion between the resin and the carrier film^21^.

Many studies have been carried out in order to understand and optimize the nanostructure design (size, shape, period, arrangement, etc.) of moth-eye inspired antireflective surfaces^22–24^. In general, these studies indicate that the nanocones should be placed at subwavelength distance to each other, and in addition, the height of the structures has to have a dimension close to that of the wavelength range to transmit. However, increasing the aspect ratio of the nanocones is detrimental for the scratch resistance and durability of the nanostructures implying severe limitations on the actual use of antireflective surfaces in real applications^4,25^.

Several approaches directed to improve the mechanical performance of the thermally nanoimprinted moth-eye nanocones have been reported. Yoo et al., coated, by atomic layer deposition (ALD), nanoimprinted PMMA and PC surfaces using inorganic materials like Al_2_O_3_^26^. Yeo et al., implemented a thermal treatment to improve the resistance of PMMA anti-reflective films^27^. In a previous work^28^, we produced surface nanocomposites of PMMA/TiO_2_ by loading the polymer surface with TiO_2_ nanoparticles prior to the thermal NIL step of the moth-eye topography. A different approach to improve the mechanical resistance, has been to modify the geometry by making pore-like inverse moth-eye nanostructures^29,30^. The improvement of mechanical resistance and the resulting optical quality, compared to moth-eye nanostructures, has been determined numerically and experimentally, and it was demonstrated that inverted nanocones were able to sustain a higher mechanical stress while retaining a good optical antireflective performance^31,32^.

In this work, R2R T-NIL is applied to produce sub-wavelength anti-reflection structures on PMMA self-standing films. In order to understand the influence of the processing parameters on the formation of the nanocone patterns and consequently, on the optical and mechanical performance of the resulting imprinted AR films, a study of the variable processing parameters, including imprint temperature and web speed, at low constant pressure is carried out. The influence of the process parameters on the geometrical characteristics and aspect ratio of the moth-eye like structures formed from protruding to intruding nanocones is examined and correlated with the optical and mechanical properties of the resulting films.

The outcome of the study in combination with finite element simulations provides guidelines for generating with a single moth-eye mold an AR topography with the desired optical performance for a given application and at the same time, predicting its mechanical robustness.

## Results and discussion

**Figure 1:**
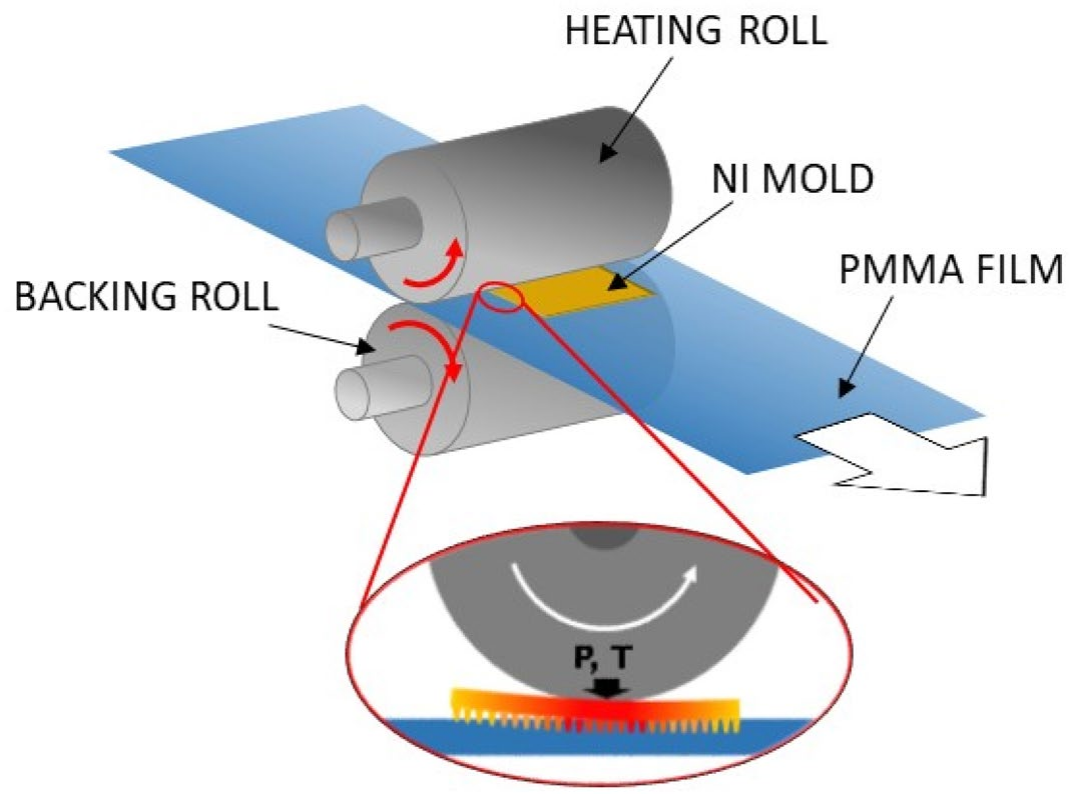
Scheme of the geometry used for the thermal R2R imprinting of PMMA self-standing film and magnification of the contact point between the roller mold and the polymer film.

The R2R T-NIL process was carried out using an electrically heated roller which was rolled over a flat nickel mold. This mold with moth-eye antireflective structures included sinusoidal 2D nanocone features with an average height of 350 nm and peak to peak distance of 290 nm. The mold was a rigid nickel sheet of 1 mm in thickness (10 × 2 cm) that was placed flat over the polymer film to be imprinted, adopting a configuration similar to that of a roll to plate system^13,33^. The configuration employed allowed to prevent film stretching of the patterned area even at the higher processing temperatures. During the process, the roll is heated and rolled over the Ni mold pressing against the PMMA film during its horizontal displacement (*cf.* Figure 1).

In thermal R2R-NIL, the key process parameters are temperature, pressure and web speed^14–16^. In this work the pressure was set to a value of 0.2–0.3 MPa for all processes. At this pressure, temperature and web speed parameters were varied to assess the pattern transfer fidelity and reproducibility. The R2R T-NIL process was carried out at temperatures ranging from 80 °C up to 140 °C, that is from below the glass transition temperature (T_g_) of PMMA (105 °C) to well above this softening temperature. The web speed was varied from 0.02 m·min^−1^ up to 1 m·min^−1^. After the process, the imprinted films were cooled down at room temperature by applying a constant nitrogen flow.

### Effect of imprinting temperature

**Figure 2:**
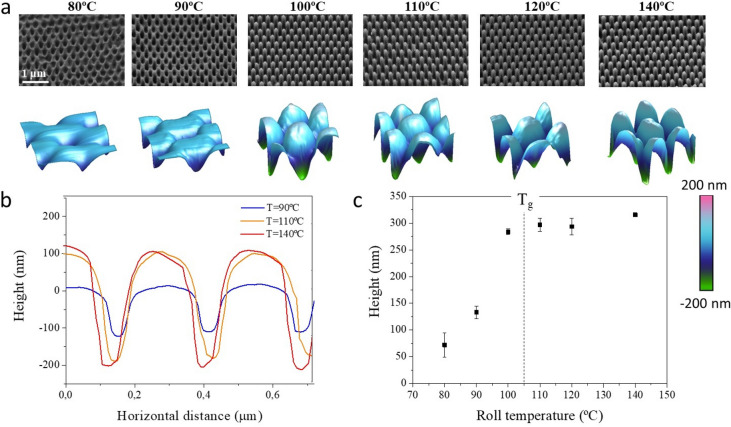
(**a**) SEM images of PMMA R2R nanoimprinted moth-eye structures fabricated at web speed of 0.02 m·min^−1^at temperatures as noted. The images were acquired at a tilting angle of 45° and at an equivalent rotation angle in all cases. The bottom row shows 3D reconstructions of AFM surface scanned images of 550 × 550 nm^2^ imprinted areas. (**b**) AFM height profiles corresponding to different processing temperatures. (**c**) Height dependence of the moth-eye nanocone structures with the processing temperature. The dotted line marks the glass transition temperature of the bulk PMMA.

At the initial experiments, nanoimprinting was carried out at two different web speeds (0.02 and 0.5 m·min^−1^) while the temperature was varied in both cases from 80 to 140 °C in steps of 10 °C. Figure 2a shows the imprinting and characterization results obtained including the SEM images and the 3D reconstruction of the AFM surface scans of the moth-eye nanoimprinted films at web speed of 0.02 m·min^−1^. It demonstrates the variation of the morphology of the moth-eye features with the processing temperature. Figure 2b shows the AFM 1D height profiles of the samples processed at the lowest, mid and highest processing temperatures. Figure 2c graphically plots the height dependence of the nanoscopic features with the processing temperature. The mean height values were calculated after measuring at least 30 individual nanocones located on different regions of the film surface. As it can be observed, the height profiles obtained can be classified into two well differentiated groups above and below the T_g_ of PMMA.

Above the T_g_, the height corresponds to that of filled moth-eye nanocone protrusions. The height and geometry seen in the SEM images agrees well with what is seen on the 3D reconstruction of the AFM surface scans.

At temperatures below T_g_, the imprinted features observed correspond primarily to inverted nanocones with a depth that increases with increasing process temperature. Below T_g_, the polymer is in its glassy state and as such, there is a high resistance of the polymer to flow. Consequently, during the imprinting process the pressure is not sufficient to squeeze the polymer into the moth-eye topography. Instead, due to the large differences in yield stress and Young´s modulus between the PMMA and the Ni mold, which is about 2 orders of magnitude^34^, permanent plastic deformation by the Ni mold nanocones is induced at temperatures below the glass transition. The resistance against deformation decreased, as the yield stress decreased with the process temperature. This can be recognized in Fig. 2c by the increase in depth of the intruding nanocones from 90 to 280 nm as the temperature is raised from 80 to 100 °C.

Investigation on the formation of cavities by a process of hot embossing below the Tg was reported by Shan and co-workers^35^. They studied the replication accuracy of the micro hot embossing process on PMMA using a Berkovich nanoindentation tip. They found that from a temperature of 85 °C (T_g_ − 20 °C) to the vicinity of the T_g_, formation of cavities was primarily attributed to plastic deformation under the nanoindentation force, and permanent cavities were formed on PMMA substrates with minor elastic recovery as the process temperatures came close to the T_g_ .

From the AFM images of the substrates imprinted below T_g_ where the inverse nanocones are produced, a material pile-up or accumulation is clearly visible around the intruding nanocones caused by the indentation on the softened polymer. This, in combination with the surface roughness of the pristine PMMA film, leads to a surface that is not completely flat but presents some inhomogeneities that will certainly have some influence on the optical and mechanical properties.

Above the glass transition, the maximum height of the nanofeatures remains approximately constant in all experiments and the topmost protruding nanocones with better replica accuracy were obtained with height values slightly above 300 nm at T_g_ + 35 °C, which is close to that of the moth-eye patterns of Ni mold (see Figure S1 in Supporting Information). This result indicates that at a temperature above T_g_, the web speed of 0.02 m·min^−1^ is slow enough to allow for the viscous polymer flow to reach a comparable level of cavity filling into the mold, rendering a mean nanocone height equivalent to that obtained under conventional thermal NIL conditions^28^.

In the next paragraphs, the performance of these films in terms of mechanical resistance and optical properties linked to the specific geometrical parameters of the imprinted nanofeatures is assessed.

**Figure 3:**
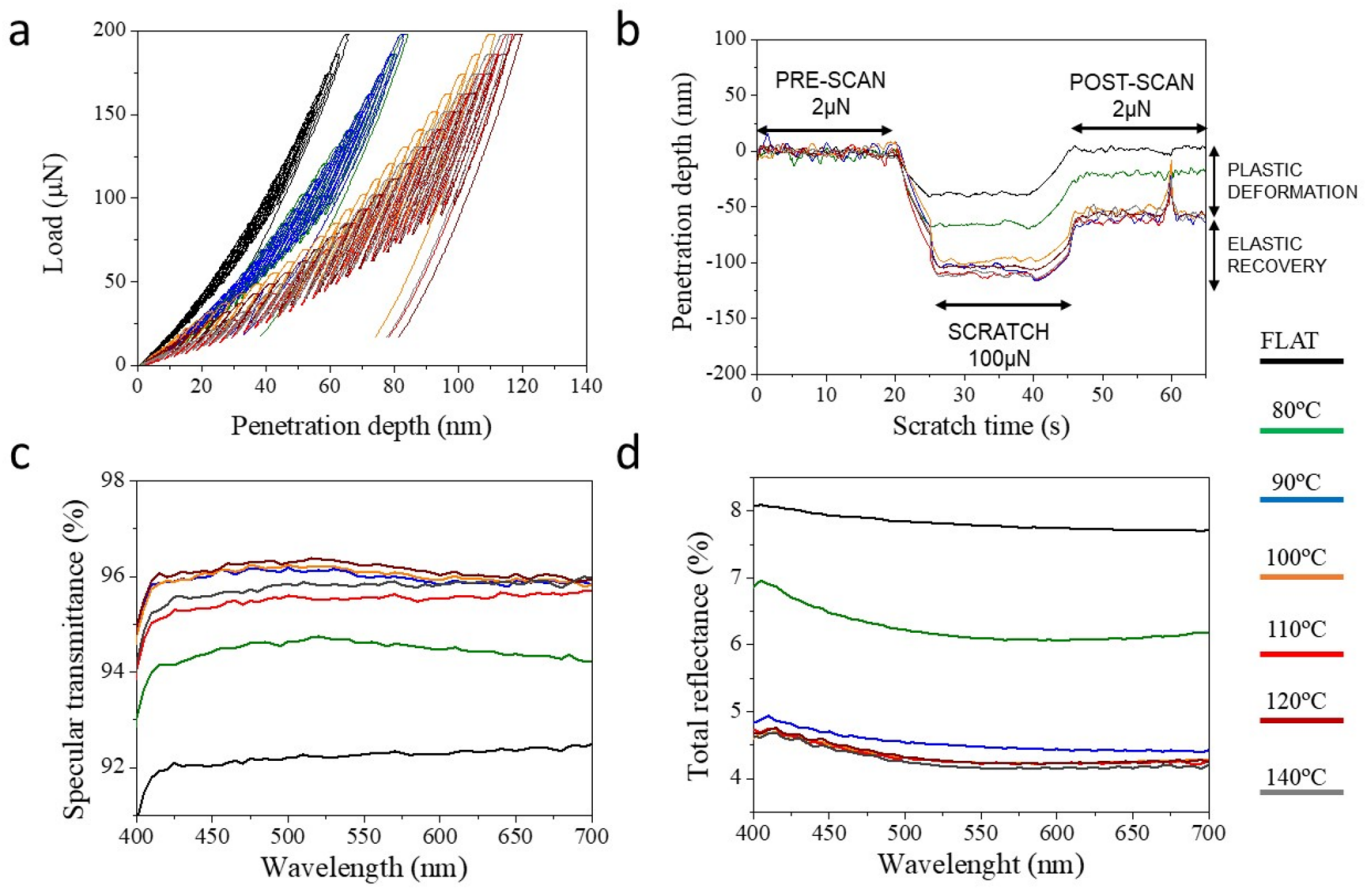
Summary of mechanical properties and broadband optical performance of the moth-eye R2R nanoimprinted PMMA films processed at web speed of 0.02 m·min^−1^: (**a**) Representative nanoindentation loading–unloading curves. (**b**) Representative nanoscratch measurements obtained for a normal applied load of 100 µN. Three regions are plotted sequentially comprising the pre-scan, the scratch and the post-scan. (**c**) Specular transmittance spectra and (**d**) total reflectance spectra.

Evaluation of the mechanical properties were carried out by nanoindentation and nanoscratch tests^28,36,37^. Figure 3a shows the characteristic curves corresponding to the load-unload nanoindentation cycles upon application of a monotonically increasing nanoindentation load up to a maximum of 200 µN on the different substrates under study. The curves represent the probe penetration depth onto the nanocone topography as a function of the load applied. Clear differences can be observed between the two groups of imprinted substrates below or above the T_g_ of the PMMA. The substrates imprinted at temperature close to the T_g_ or higher, that is with protruding nanocones, presented higher penetration depth values for the same applied load (up to 115 nm at max. load), in other words, a lower resistance to probe indentation compared to those imprinted below the T_g_ where the nanocones were intruding._._ On the other hand, it can be appreciated that the substrates imprinted below T_g_ at 80 and 90 °C, show a more elastic behavior whereby the loading and unloading curves exhibited less hysteresis. On these substrates, the probe penetration (80 nm ) was close to that of the flat substrate (60 nm).

Nanoscratch tests displayed the same trend. Figure 3b shows the characteristic nanoscratch measurements comprising three different scans: pre-scan, scratch and post-scan. Pre and post imaging scans were accomplished at a small contact load (*ca.* 2 µN) while the scratch was performed at 100 µN. The nanoscratch probe performs forwards/backwards cycles over the same track but, for the sake of clarity, the depth penetration of each trace is represented in sequence, as a function of the time taken for the scan. The load profile and probe lateral displacement are included in the Supporting Information (Figure S2) for clarification of the coupling between probe displacement and applied force during the test. The results provide information about the residual plastic deformation caused by the scratch, which is directly related to the probe footprint, i.e., the post-scan penetration depth. The elastic recovery is the difference between the maximum probe penetration during the scratch, and the residual penetration depth measured during the post-scan step. The films imprinted above T_g_, having the full moth-eye texture, presented a plastic deformation close to 50 nm in all cases. In comparison, substrates processed below the T_g_ showed a lower residual deformation. Specifically, the plastic deformation observed on the substrate prepared at 80 °C was 20 nm.

The optical characterization of the nanoimprinted samples was performed by using a UV–Vis spectrophotometer with integrating sphere, as described in the experimental section. Figure 3c,d summarizes the optical performance of the different imprinted films. The specular transmittance and total reflectance curves obtained from nanoimprinted films are compared to that of the PMMA flat film as reference. In general, an improvement on transmittance and reflectance is observed upon imprinting of the PMMA film. The values obtained can be classified in two different groups. The moth-eye like nanostructure, obtained upon nanoimprinting at higher temperatures, shows better performance in all cases. Nevertheless, the films imprinted with intruding moth-eye features and processed below T_g_, presented also an enhanced optical behavior while holding good mechanical properties, as noted before. For instance, the film processed at 90 °C shows a 3% improvement in transmittance compared to a non-textured flat film.

**Figure 4:**
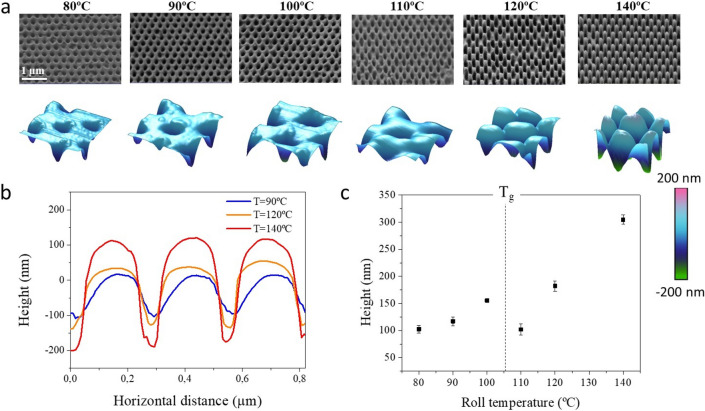
(**a**) SEM images of PMMA R2R nanoimprinted moth-eye structures fabricated at web speed of 0.5 m·min^−1^ and different temperatures as indicated. SEM images were acquired at a tilting angle of 45° with an equivalent rotation angle in all cases. The bottom row shows 3D reconstruction of AFM height images of areas *ca*. 550 × 550 nm^2^. (**b**) AFM surface profiles corresponding to selected substrates processed at the indicated temperatures. (**c**) Height dependence of the nanofeatures with processing temperature.

The impact of a higher processing speed on the imprint results was next investigated. The web speed determines the contact time between mold and polymer film, this is the instant when heat and pressure are applied from the roller-mold to the polymer film at the point of contact (See Fig. 1). Hence, the amount of heat transfer and the pressure driven viscous polymer flow into the mold cavities directly depend on this contact time. Figure 4a shows the SEM images from the films prepared at web speed of 0.5 m·min^−1^, that is, 25 times faster than the previous experiment, together with the 3D reconstruction of the AFM scanned topographies obtained. In this case, a more gradual transition from a topography of intruding nanocones to a topography of protruding nanocones can be appreciated. The 1D height profile extracted from selected films is shown in Fig. 4b. The nanocones height dependence with the processing temperature is presented in Fig. 4c. In this case, a quasi linear dependence of the height is observed in both groups below and above the PMMA T_g_. Nonetheless, a smaller than expected nanocone mean height was observed on films processed at 110 °C, that is slightly above the Tg compared to that obtained at 100 °C. Similar anomalous non-linear behavior has been observed previously while performing a study on PMMA hot embossing processability within a range of temperatures across the Tg region^38^. In this work, it was shown that the PMMA behavior changed abruptly at temperatures from the T_g_ to around 10–20 °C above the T_g_. Within this temperature range, PMMA polymer chains entanglements allow for a large stress holding capability and, consequently, a larger extent of the instant and retarded elastic recovery components upon load release, causing a larger reduction of the embossed features than that achieved at temperatures beyond this range.

This anomalous behavior was found somehow more apparent at the faster web speed of 0.5 m·min^−1^ than in the previous experiments at 0.02 m·min^−1^, indicating that the elastic recovery is larger at the faster processing speed. Rationalization of this trend can be found on the shorter time for stress relaxation during the imprint step which leads to a lower reduction in elastic stress and a larger recovery after the force is released^35^.

It is worth to note than in the present study, the morphological characterization was accomplished at least 1 week after sample processing so that, the relaxation effects have been accounted for.

**Figure 5:**
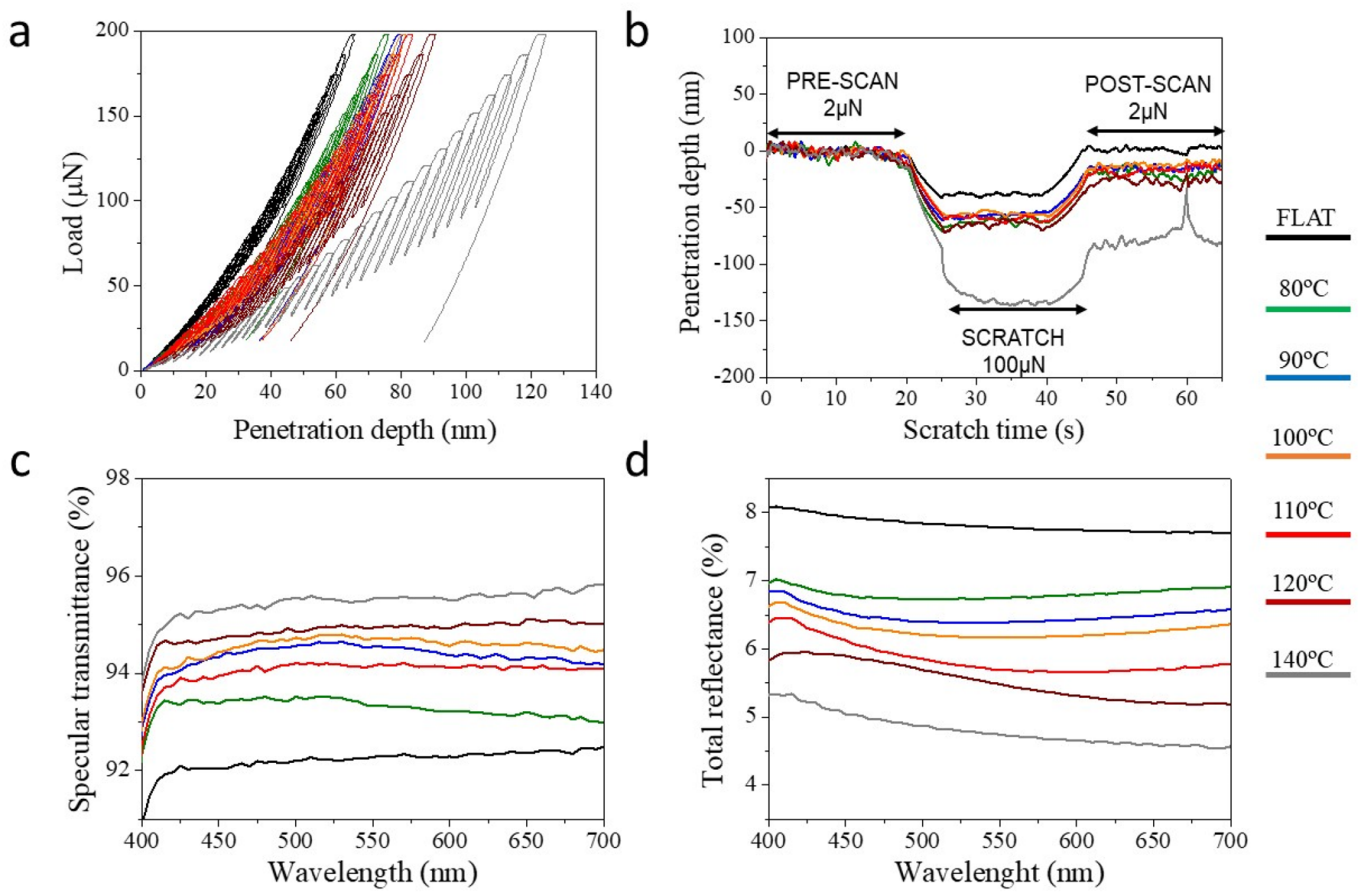
Summary of mechanical properties and broadband optical performance of PMMA moth-eye R2R nanoimprinted films processed at web speed of 0.5 m·min^−1^: (**a**) Representative nanoindentation loading–unloading curves. (**b**) Representative nanoscratch measurements obtained for a normal applied load of 100 µN. Three regions are plotted sequentially comprising the pre-scan, the scratch and the post-scan. (**c**) Specular transmittance spectra and (**d**) total reflectance spectra.

Figure 5 summarizes the mechanical and optical performance of films imprinted at different temperatures for a web speed of 0.5 m·min^−1^. Compared to those samples prepared at equivalent temperatures but using a lower web speed ( see Fig. 3), it can be observed that the nanoindentation curves are shifted, in each case, to lower probe penetration values, indicating a higher resistance to deformation. Only the film printed at 140 °C exhibited similar penetration depths to that printed at lower web speed, while for the films processed at all other temperatures, the penetration depths were < 90 nm. A similar trend is observed in the nanoscratch measurements, showing decreased plastic deformation values, which in some cases can be as low as 10 nm. The degree of plastic deformation has in fact a direct relationship to the aspect ratio of the moth-eye topography being in this case much reduced and predominately formed by inverted nanocones. Consequently, the scratch resistance of the films is close to that of pristine flat films. Only the substrates processed at the higher temperature (140 °C) present values comparable to those obtained at lower web speed.

Figure 5c,d present the optical transmittance and reflectivity of the imprinted films, showing a gradual improvement from the flat PMMA to the nanoimprinted films at 140 °C, in accordance to the gradual increase in aspect ratio of the moth-eye nanocones. The optimum values obtained for transmittance (*ca*. 96%) and reflectance (*ca.* 4.5%) are similar to those observed on substrates processed at lower velocity.

**Figure 6:**
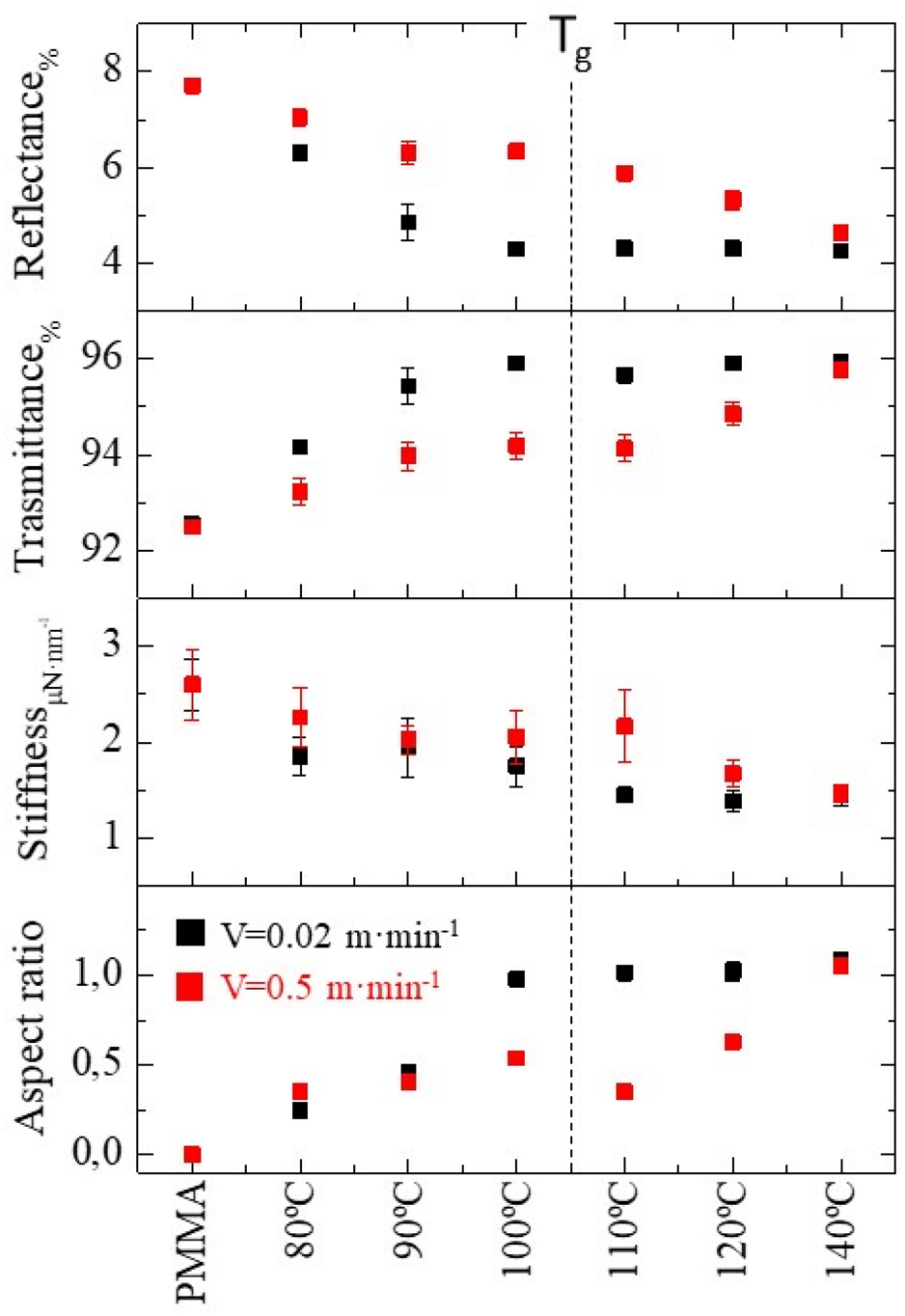
Summary of the R2R nanoimprinted moth-eye films characteristics related to topography aspect ratio, optical and mechanical performance as a function of the processing temperature obtained at web speed of 0.02 m·min^−1^ (black) and 0.5 m·min^−1^ (red). The data corresponding to the pristine PMMA film are included for comparison. The dotted line marks the PMMA T_g_.

In order to find a correlation between the processing conditions and the mechanical and optical properties of the imprinted films, in Fig. 6 several film properties are plotted as a function of the fabrication parameters. The aspect ratio of the moth-eye features was obtained from the AFM scanned images. This value was estimated as the ratio between the maximum height/depth measured divided by the peak to peak distance of the hexagonal nanofeatures array.

The comparison of the mechanical properties between the reference PMMA pristine flat film and the nanoimprinted films was done on the basis of the “contact stiffness”, defined as the rate of change of probe penetration depth with load within the elastic nanoindentation regime which in this case corresponds to probe penetration depth below 10 nm. In terms of optical properties, the transmittance and reflectance values at 700 nm are included. These data obtained at both processing velocities: 0.02 and 0.5 m·min^−1^, are plotted concurrently in Fig. 6. Figure 6 allows visualizing the direct relation between processing parameters and film performance. As expected, better optical behavior is achieved for the moth-eye structures with the higher aspect ratio in agreement with what has been theoretically predicted and experimentally observed in previous works^39^. It can be seen that for the same process temperature, the aspect ratio of the moth-eye topography fabricated at reduced web speed is higher, providing better optical performance. Transparency is increased while total reflectance decreases down to 4%. However, higher aspect ratio structures show poorer mechanical resistance, as it can be inferred from the decrease of the apparent stiffness by 50%, compared to the flat PMMA film. For a web speed of 0.02 m·min^−1^, there exists a relatively narrow processing window at temperatures slightly below the T_g_ of the polymer matrix (~ 100 °C), where a good compromise for both optical performance and mechanical resistance can be seen when films exhibit reflectance values as low as 4% and plastic deformations below 20 nm. At this processing conditions, low aspect ratio nanoscopic features allow minimizing the decrease in the apparent stiffness while, at the same time, the optical response is within the optimal achievable range. When processing at a web speed of 0.5 m·min^−1^, it is necessary to shift the processing temperature to higher values, above 120 °C, to obtain equivalent results.

### Effect of web speed

Next, the effect of the web speed, at constant temperature, was investigated as it is one of the most critical parameters affecting the pattern transfer during thermal R2R-NIL processing^40^. It was previously mentioned that the web speed defines the contact time between mold and the polymer. As such, the contact time determines the amount of heat transfer to the polymer substrate increasing the chain mobility and so, its viscosity. This in turns determines the viscous flow rate into the mold cavities driven by the pressure applied, and consequently the degree of filling of the mold and the height of the imprinted features. The dependence of nanofeature height with imprint time has been studied theoretically^41,42^.

In thermal R2R-NIL, temperature and web speed are indeed interrelated parameters and need to be adjusted concurrently. As previously noted, too high temperatures may cause the polymer web film stretching under the roller tension causing pattern distortion and consequently, it is imperative to reduce the processing temperature as much as possible.

Hence, based on the experiments described above the temperature of 110 °C, slightly above the T_g_, was selected as best suited temperature. Fixing the roll at this temperature, the effect of the web speed on the formation of the moth-eye structures was investigated.

Likewise, from the industrial production point of view, it is also of high interest to reduce the processing temperature and to have extremely short processing times by increasing web speed to the maximum.

**Figure 7:**
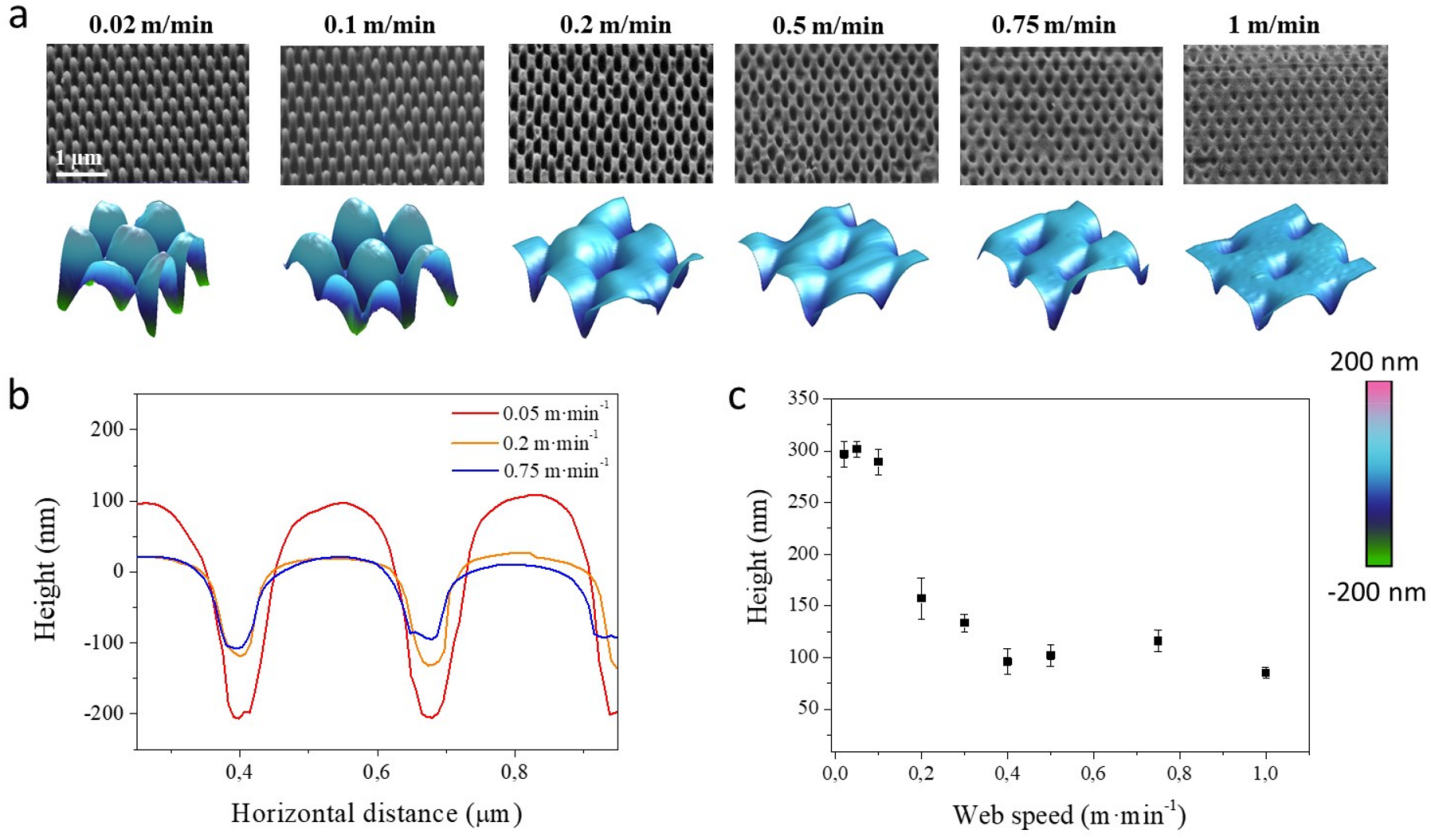
(**a**) SEM images of PMMA R2R nanoimprinted moth-eye structures fabricated at constant roll temperature (T = 110 °C) and at different web speeds as noted. SEM images were acquired at a tilting angle of 45° with an equivalent rotation angle in all cases. Bottom row shows the 3D reconstruction of AFM scans of 550 × 550 nm^2^ areas. (**b**) AFM height profiles corresponding to selected processing temperatures. (**c**) Height dependence of the nanoscopic features with processing speed.

Figure 7a shows SEM images of the nanoimprinted films when the imprinting web speed was varied from 0.02 up to 1 m·min^−1^ together with the corresponding 3D AFM images of the obtained imprinted films. Figure 7b shows 1D height profiles extracted from selected films.

It can be observed in Fig. 7c that as the web speed is increased, the aspect ratio of the protruding moth-eye nanocones is reduced, and at high speed above 0.2 m·min^−1^, only intruding nanocones are obtained.

As mentioned above, this is a direct result of the reduction of the contact time at faster process speed. Additionally, Mäkëla et al.^40^, reported an additional effect of the process speed. They observed a reduction on the process set temperature at the surface of the Ni mold due the cooling effect from the web moving at higher printing speeds. Hence, both effects converge, on one hand, the reduction of temperature and associated polymer viscosity and on the other hand, the reduction of time for the polymer to flow into the mold cavities. Accordingly, as we see on these experiments, at higher web speeds only intruding nanocones with similar depth are obtained.

**Figure 8:**
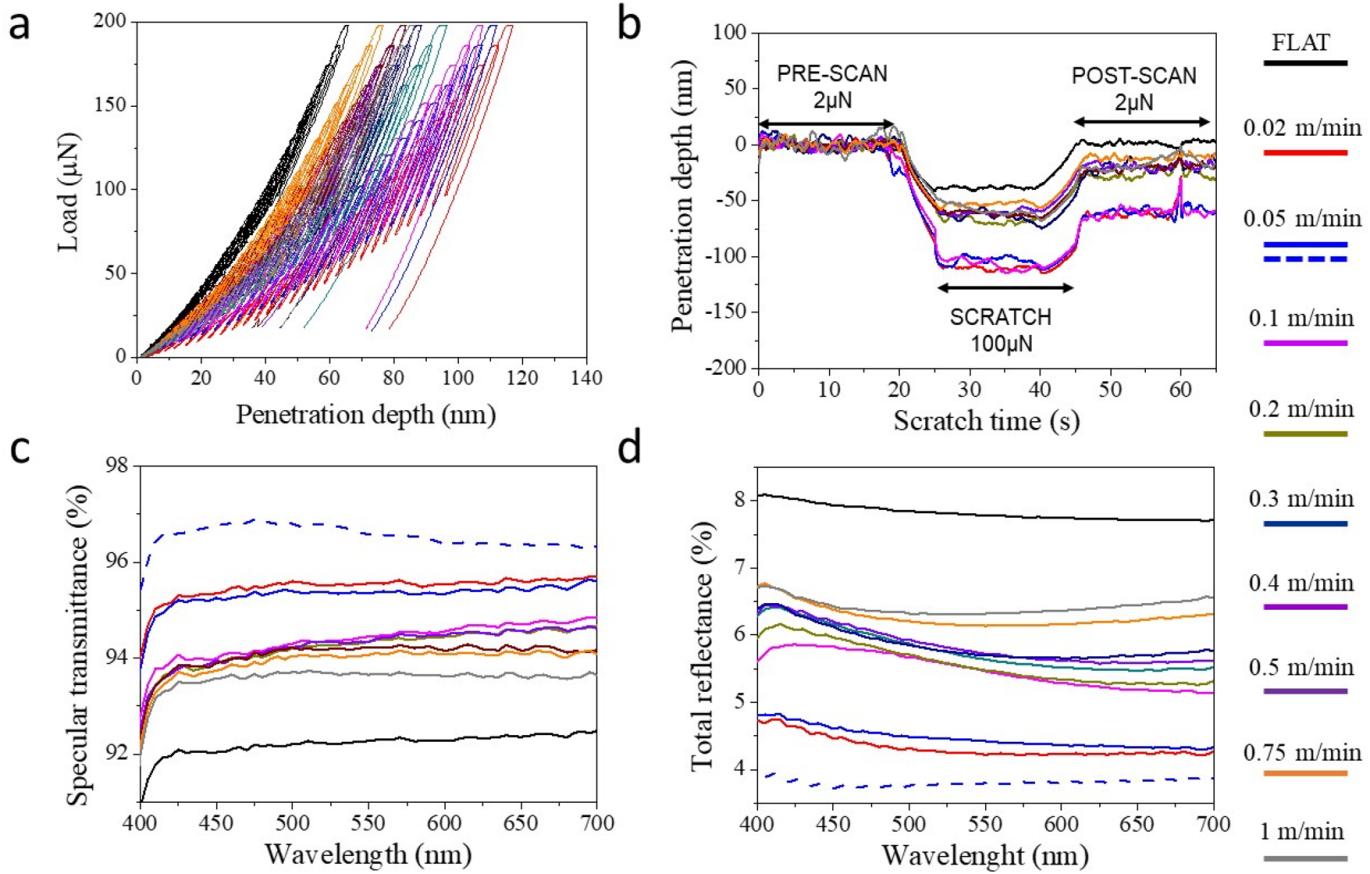
(**a**) Representative nanoindentation loading–unloading curves obtained for the nanoimprinted moth-eye films processed at 110 °C and at different web speeds. (**b**) Representative nanoscratch measurements obtained for normal applied load of 100 µN. Broadband optical performance of the R2R nanoimprinted substrates: (**c**) specular transmittance spectra and (**d**) total reflectance spectra. The dotted line corresponds to a two-sided nanoimprinted moth-eye PMMA film prepared at 110 °C and 0.05 m·min^−1^.

The evaluation of the mechanical properties of nanoimprinted moth-eye structures processed at 110 °C and at different web speeds in terms of mechanical resistance is shown in Fig. 8a, where characteristic nanoindentation load-penetration curves are displayed. The graph shows that the mechanical response is well correlated with the nanostructure height whereby, lower probe penetration for equivalent loads are observed for lower aspect ratio structures and intruding nanocones. Substrates prepared at low speed (below 0.2 m·min^−1^) where the aspect ratio is highest, show higher probe penetration (up to 120 nm) for an equivalent applied force (up to 200 µN) in comparison to those prepared above such web speed, for which the maximum penetration depths decreased with increasing web speed. These results indicate that the fully developed high aspect ratio moth-eye like topography offers the lower mechanical resistance whereas the substrates prepared at web speeds above 0.5 m·min^−1^, exhibit the lowest penetration depths, hence, the highest mechanical resistance, only *ca.* 10% lower than that measured on the reference flat PMMA film. The nanoscratch tests are in good agreement with this trend. The plastic deformation observed on the moth-eye surfaces upon performing the scratch tests presents a similar tendency. Nanoimprinted substrates fabricated at lower web speeds show the higher plastic deformation (above 50 nm) while the films produced at web speed over 0.2 m·min^−1^ reach values not superior to 25 nm (*cf.* Figure 8b).

Similar to that observed for substrates processed at constant velocity of 0.5 m·min^−1^, the optical performance of the films gradually improved upon texturization in all cases. The transmittance of the films increased after texturization, from 92% up to more than 95%, while the total reflectance decreased from 8% down to 4%, as it can be observed in Fig. 8c,d.

**Figure 9:**
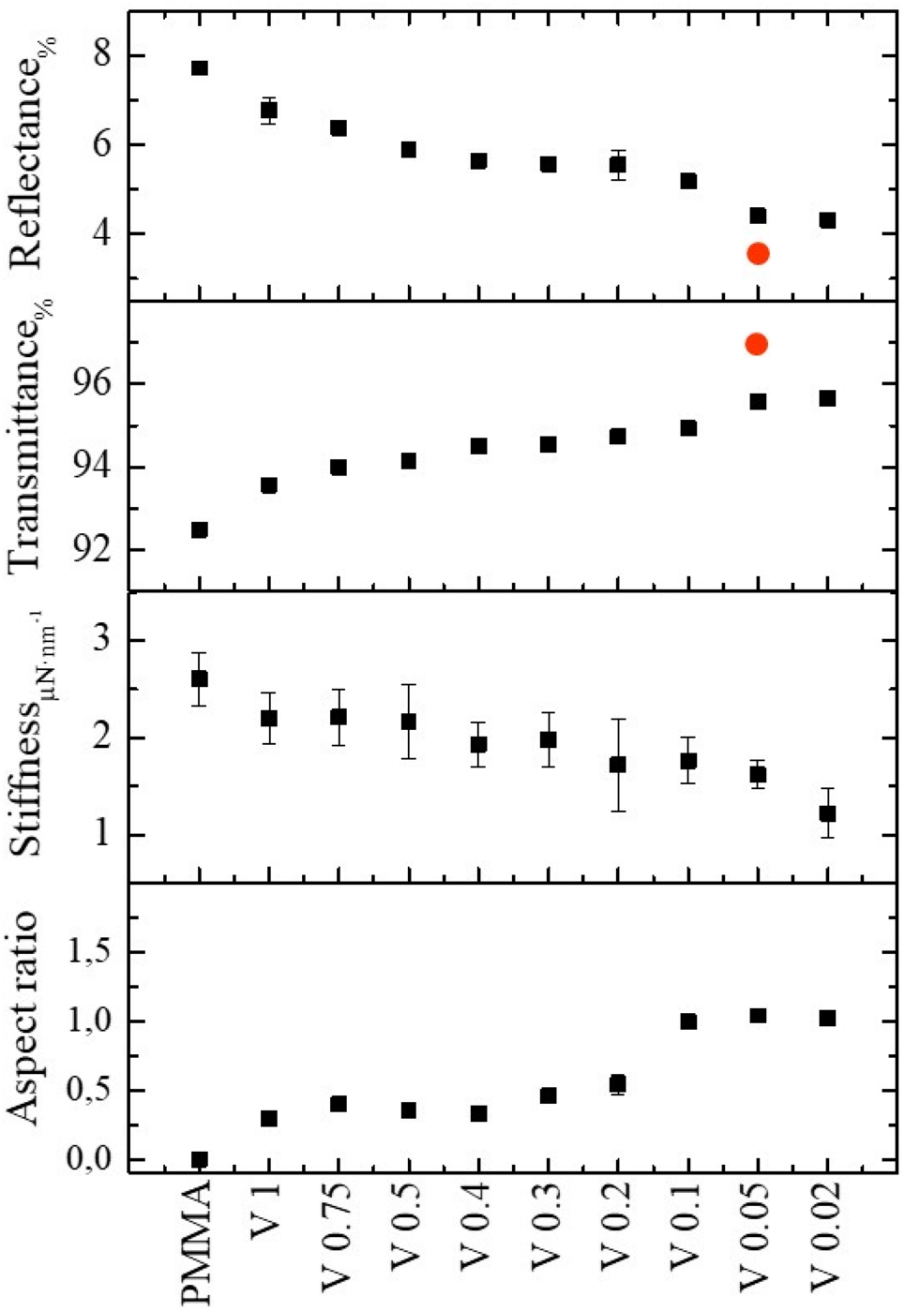
Summary of the R2R nanoimprinted moth-eye films characteristic parameters related to substrate topography, optical and mechanical performance as a function of the processing web speed obtained at constant roll temperature of 110 °C. Red circles correspond to the values obtained from two-side nanoimprinted PMMA film.

Figure 9 summarizes the mechanical and optical performance together with the topography aspect ratio imprinted at a constant temperature and at variable web speed as described above. The graph illustrates the correlation among them, with the gradual trends observed, signifying the direct influence of the web speed on the pattern transfer during roll to roll processing.

All the given transmittance and reflectance values are obtained from PMMA films imprinted on a single side. When the moth-eye structures are produced on both sides of the film, the optical performance substantially improves as expected. Mäkelä et al*.* demonstrated the feasibility of performing two-sided thermal R2R-NIL on thermoplastic polymer films using both sequential and simultaneous nanoimprint approaches^43^. In their work, a simultaneous approach was implemented whereby a free standing PMMA film was placed between two Ni molds, and the assembly was subsequently heated and rolled-pressed during the horizontal roll displacement. Due to tool constrains, only the top roller was heated during the process, however, the pressure and heat transfer to the bottom side of the PMMA film was sufficient to allow for the imprinting of the bottom part of the film.

The optical properties of a two-sided nanoimprinted film fabricated at 110 °C and 0.05 m·min^−1^ are included in Fig. 8c,d (dotted lines) and Fig. 9 (red circles) for ease of comparison. This substrate showed the best transmittance (~ 97%) and reflectance (˂ 4%) values.

### Numerical simulation of mechanical behavior and dependence on nanocone geometry

**Figure 10:**
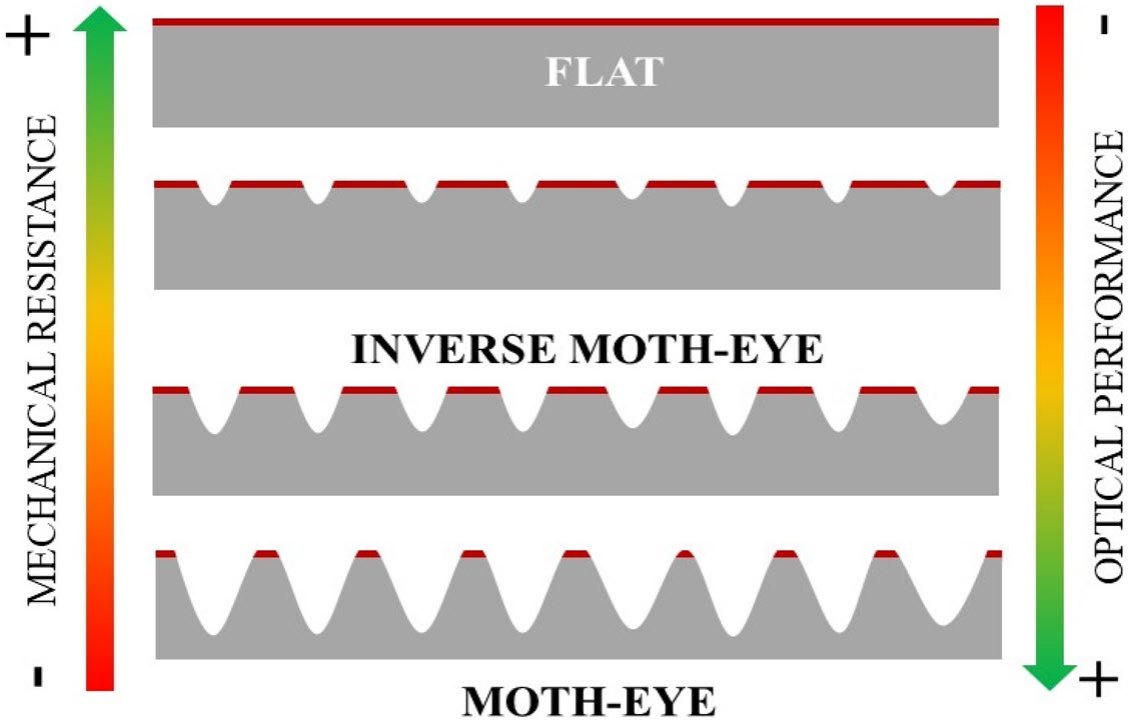
Schematic view of the nanostructure evolution during thermal R2R-NIL from flat PMMA to intruding nanocones and to fully developed moth-eye structure showing a reduced contacting surface (red lines in each case).

Figure 10 illustrates schematically from top to bottom the moth-eye nanostructure evolution from intruding nanocones to protruding moth-eye like nanostructures with decreasing processing web speed (at constant temperature) and increasing temperature (at constant web speed). The impact on the film mechanical and optical properties is indicated by the arrows. The practical implication is that inverted nanocones are able to sustain higher mechanical stresses, while still retaining a significant antireflective optical performance, as has been proposed before^29–32^.

Qualitatively, this geometrical effect on mechanical performance can be ascribed to the reduced surface contact as the moth-eye nanocones develop, represented by a red line in Fig. 10. In order to get a better understanding and to guide the design of mechanically robust antireflective surfaces, the nanoindentation response of a flat surface, intruding nanocones and protruding moth-eye nanocones were simulated by the Finite Element method (FEM). The details of the numerical simulations are described in the experimental methods section. The dimensions of the intruding and protruding nanocones were chosen in order to approximate as close as possible to the real dimensions of the nanotopography obtained at web speed of 0.02 m·min^−1^ and processing temperatures of 80 and 140 °C respectively. As seen on the AFM images of Figs. 2a and 4a, the inverse moth-eye topography produced below Tg shows an irregular surface consequence of the original roughness on the PMMA film and the material pile-up during the imprinting of the nanocones. Hence, since these inhomogeneities can affect the mechanical response of the topography, to quantify for their effect on the mechanical performance, a second model was created whereby small protrusions representing the roughness of the flat areas surrounding the structures were added to the inverted nanocone topography with the same geometry modeled before (see numerical methods section).

For the simulation of the nanoindentation tests, the geometries considered were flat, intruding (with and without considering roughness) and extruding. Details are given in the numerical methods section. Table 1 shows the simulation and experimental conditions for the nanoindentation tests, along with the maximum displacements of the tip (at the maximum load of 200 μN) and relative errors.

**Table 1:** Simulation and experimental conditions of the nanoindentation tests, showing maximum depths and relative errors.

Experimental condition	FEM simulation description	Maximum depth in experiment (at 200 µN) [nm]	Maximum depth in simulation (at 200 µN) [nm]	Relative error (%)
Flat PMMA	Flat domain (Fig. 12a)	65.2	63	3.4
Nanoimprinted PMMA processed at **80 °C** and web speed of **0.02 m/min**	Intruding geometry + idealized roughness (Fig. 12d)	83.4	85	1.9
Nanoimprinted PMMA processed at **80 °C** and web speed of **0.02 m/min** + **15 µN pre-indentation** to eliminate roughness	Intruding geometry (Fig. 12c)	61.6	66	7.1
Nanoimprinted PMMA processed at **140 °C** and web speed of **0.02 m/min**	Protruding geometry (Fig. 12b)	113.9	114	0.1

**Figure 11:**
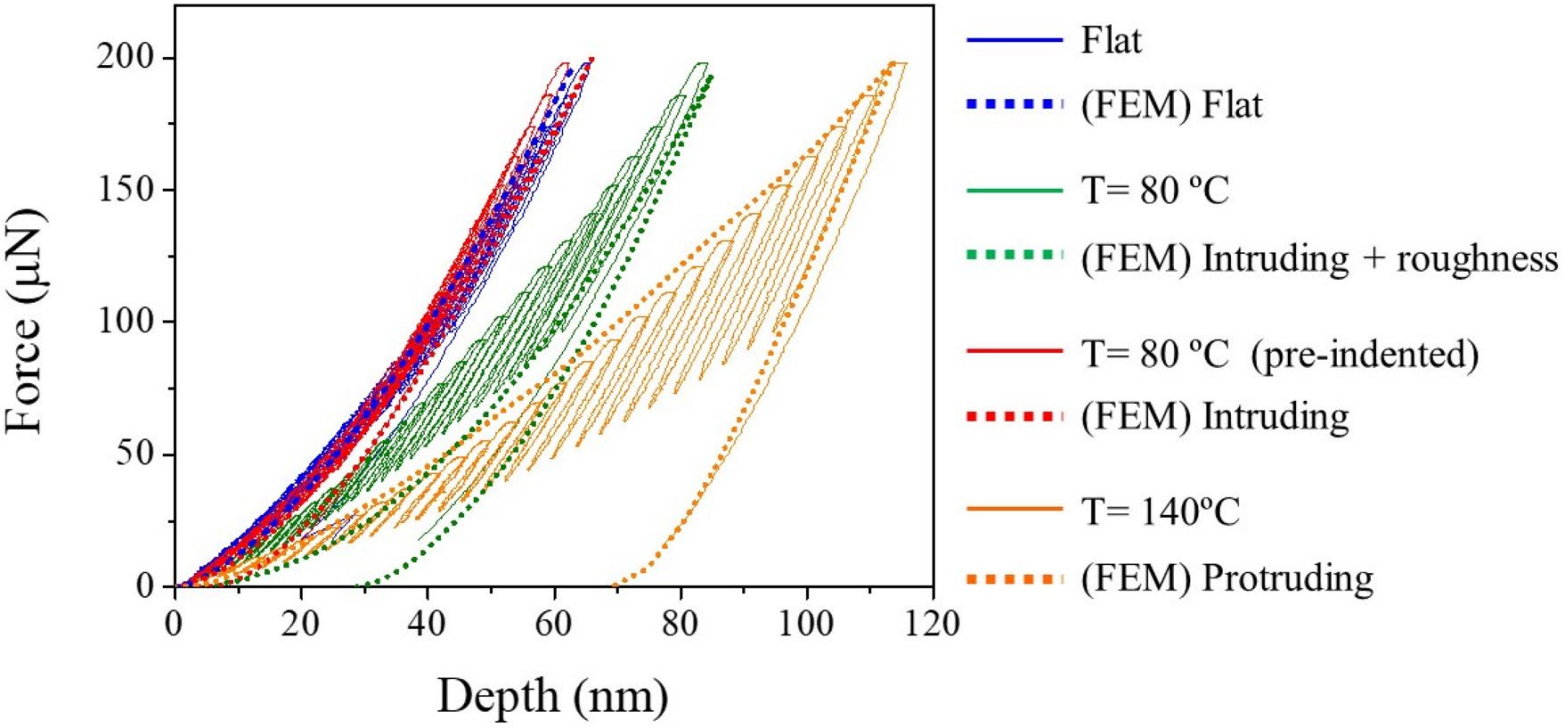
Finite element simulated load–displacement curves (dotted lines) obtained for the flat, inverted nanocone (with and without roughness) and protruding moth-eye nanostructure. The simulated curves are compared with the experimental load–displacement curves (continuous lines) of the corresponding nanostructures obtained at different temperatures and web speed of 0.02 m·min^−1^. In the case of the sample processed at 80 °C a curved obtained after sample preindentation is additionally included.

Figure 11 shows the simulated load–displacement curves obtained for the flat, intruding and protruding nanocone topographies, compared with the experimental load–displacement curves. The good correlation between the experimental and simulated curves shown for the flat surface, both with maximum penetration depths of ~ 63–65 nm, verifies the numerical simulations and the right choice of the material parameters for PMMA. For the protruding moth-eye surface topography, the deformation behavior was also accurately predicted by the simulation, with a maximum penetration depth of ~ 114 nm in both cases. This confirms that the material properties do not change upon thermal NIL processing, which was an implicit assumption of the numerical simulations. The agreement is also particularly good for the intruding geometry, provided that the surface roughness is taken into account, with both the experimental (T = 80 °C) and simulated (FEM intruding + idealized roughness) curves reaching penetration depths of the order of 83.4 and 85 nm respectively.

The later can be further confirmed by designing one specific nanoindentation measurement. The simulations also allowed elucidating the impact of the roughness on the mechanical response of the inverted nanocones. In a final experiment, taking advantage of the high positioning precision of the sample stage, the inhomogeneities inherent to the processing conditions of the intruding geometry (obtained at T = 80 °C and v = 0.02 m·min^−1^) were eliminated by pre-indenting the top surface with a low force of ~ 15 µN, just enough to “flatten” the surface roughness. Then the nanoindentation tests were performed on the same site,. The experimental indentation curve after “pre-indentation” of the inverted moth-eye nanostructure (*cf.* Figure 11 red line, T = 80 °C pre-indented) fits well with the simulated curve without considering roughness (dotted red line, FEM intruding), and reflects a similar mechanical behavior to that of the flat surface. This result indicates that elimination of the surface inhomogeneities results in a mechanical response that is almost identical to that of the flat surface. Hence, the predictions of the mechanical robustness of the antireflective surfaces obtained are in good agreement with the experimental results.

Finally, in order to complete the characterization, the wetting behavior of the processed films was evaluated by static water contact angle measurements (WCA). Figure S3 shows the WCA values of the films fabricated at 110 °C at different web speeds. Yet again, a clear dependence of the wettability of the films with the processing conditions is observed. Compared to the flat PMMA all nanostructured films present larger WCA values, up to more than 150°, for the fully developed moth-eye like texture, which indicates that the films produced are highly hydrophobic.

## Conclusion

The feasibility of using thermal R2R-NIL technology to produce antireflective moth-eye like surfaces directly on free standing thermoplastic PMMA films has been demonstrated.

A systematic investigation was provided on the influence of the web speed and temperature processing parameters on the formation of the antireflective topography.

The results indicate that web speed and temperature have a profound effect on the aspect ratio of the imprinted antireflective features and consequently on the optical and mechanical performance of the films. It was seen that the aspect ratio produces in fact opposing effects on these two properties.

The finite element simulations model proposed was able to correctly describe the mechanical behavior of the moth-eye PMMA imprinted nanostructures. The mechanical behavior observed for the different antireflective topographies was due purely to geometrical factors.

A quantitative correlation including the process parameters with the optical and mechanical properties of the films has been established to serve as design guideline for selecting a suitable combination of process parameters to produce antireflective films with the desired optical performance and mechanical stability.

### Experimental methods

Roll to roll thermal nanoimprint lithography was performed using a R2R nanoimprinting tool (PTMTEC). PMMA self-standing web 10 cm wide, 275 μm thick film (Evonik Industries AG) was employed. Web tension (60–65 N) and heating roll pressure (0.2–0.3 MPa), controlled by an air cylinder were kept constant throughout all experiments. The nickel moth-eye antireflective mold (HT-AR-02) was obtained from Temicon.

SEM characterization was performed using a FSEM microscope (Auriga, Carl Zeiss) working at low voltage and current (1KV, 10 pA). AFM was performed in tapping mode using a Bruker Nanoscope 8 Multimode. The tip (Tap300GB-G, Budget Sensors) geometry allowed to obtain reliable information about the height of the nanoscopic features. All the scans were performed in the same direction relative to the sample orientation to avoid tip artifacts and obtain comparable height values.

For the mechanical characterization, nanoindentation and nanoscratch tests were performed using a Hysitron TI-950 TriboIndenter. A spherical diamond probe (radius of 10 μm) was employed for both types of measurements. During the nanoindentation tests, the force and penetration depth of the probe, driven into the sample surface, are measured during multiple “load-hold-unload” cycles up to the maximum load^44^. In this case, the measurements consisted of 20 load-hold-unload cycles keeping segment times constant (*ca*. 1 s). An unloading percentage of 50% of the maximum load for each cycle was set until a maximum final load of 200 μN was reached. A total of 20 indentations were carried out per substrate and at least two different substrates were tested for each processing condition. Nanoscratch tests were performed by moving the probe laterally. At least 10 indentations were carried out per substrate and at least two different substrates were tested for each processing condition. A typical nanoscratch testing consists of scratches of 16 µm in length using a constant normal force of 100 µN. Pre and post scans with a low normal force of 2 µN were performed in order to obtain line scans of the topography of the scratched area before and after the test, allowing to estimate the plastic deformation remaining on the nanoimprinted substrate.

Optical characterization regarding specular transmittance and total reflection measurements were performed by using a Lambda 950 UV–Vis spectrophotometer (Perkin Elmer) fitted with a 150 mm integrating sphere. Both parameters were recorded in the range from 400 up to 1200 nm. The reading area was estimated to be *ca*. 1 cm^2^. Each sample was measured at least in two different positions.

Wetting properties were studied by performing static water contact angle measurements using an optical tensiometer (Attension Theta, Biolin Scientific). Water drops of 2.5 μL were gently deposited onto the substrates. At least five different areas were measured in each sample to obtain reliable WCA values. Prior to the contact angle characterization, a silane monolayer based on FDTS (1H, 1H, 2H, 2H-Perfluorododecyl-trichlorosilane, Alfa Aesar) was deposited in gas phase, using the procedure described by Z. Pan et al.^45^*.*

### Numerical methods

**Figure 12:**
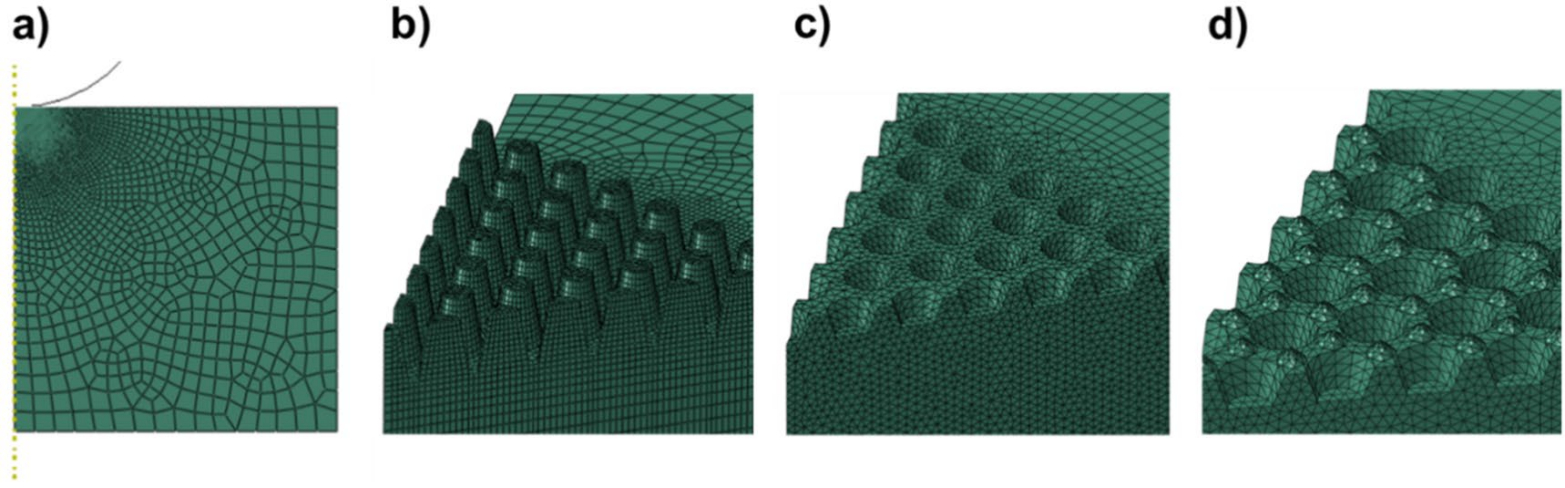
Simulated nanostructured surfaces: (**a**) flat surface, (**b**) moth-eye nanostructure (**c**) inverted nanocone with flat surface and (**d**) inverted nanocones including roughness and material pile-up at the sides.

The spherical nanoindentation of different nanostructured surfaces was simulated using the commercial FEM software Abaqus®. The Abaqus implicit solver was used in all cases. Two types of models were constructed. The flat surface was modeled as a 2D domain by assuming radial symmetry, as shown in Fig. 12a. The rest of nanostructured surfaces were modeled in 3D, considering only one sixth of each domain to take advantage of the hexagonal symmetry of the problem. For this, symmetry boundary conditions were applied to the two lateral boundaries of the domains, while the displacements were constrained at the bottom of the domain. The moth-eye nanostructure consisted of tapered pillars with base and top diameters of 250 nm and 150 nm, respectively, and a height of 350 (Fig. 12b), which corresponds to the film processed at v = 0.02 m·min^−1^ and T = 140 °C. The inverted nanocones (Fig. 12c) were modeled with tapered holes of top and bottom diameters of 230 nm and 150 nm, respectively, and a height of 120 nm depth (Fig. 1c), which corresponds to the film nanoimprinted at v = 0.02 m·min^−1^ and T = 80 °C. Since the inverted nanocones manufactured below T_g_, showed, surface inhomogeneities, to quantify its influence on the mechanical performance, the inverted nanocone topography was modeled with the same geometry as above but additionally, including small protrusions on the flat surfaces surrounding the hole representing the inhomogeneities due to roughness and material pile-up at the nanocone sides (Fig. 12d). These protrusions had a base and top diameters of 100 nm and 20 nm, respectively, and a height of 30 nm. The indenter was modeled as a rigid analytical solid with frictionless contact. The nanoimprinted surfaces were modeled as deformable bodies and meshed with CAX4 elements for the axisymmetric models, and with C3D6 and C3D8 elements for the 3D domains. The area beneath the indenter was meshed with finer elements that get progressively coarser far away from the contact area. The indentation experiments were carried out by applying a constant displacement rate scheme to the rigid indenter to a maximum load of 200 μN, followed by a reversed displacement scheme until total unloading.

An isotropic linear elastic material with Drucker-Prager plasticity and no hardening was used to model the PMMA. The Drucker-Prager plasticity captures the typical pressure sensitivity of polymers. Four parameters are required to define the material: elastic modulus, Poisson’s ratio, yield stress and frictional angle, which were set to the values shown in Table 2, by fitting the indentation response on the flat surface. These values are in good agreement with the properties expected for PMMA^46,47^. The same material parameters were used in all cases, which implicitly indicate that the material properties do not change upon thermal NIL processing.

**Table 2:** Material parameters for material model in FEM simulations.

Elastic modulus	Poisson’s ratio	Yield stress	Frictional angle
2.4 GPa	0.4	80 MPa	20°

## Supplementary Information

Supplementary Information
