# Supplementary material for: Bioinspired antireflective flexible films with optimized mechanical resistance fabricated by roll to roll thermal nanoimprint
Source: Sci Rep. 2021 Jan 28;11:2419. doi: 10.1038/s41598-021-81560-6 (PMC7844253; doi:10.1038/s41598-021-81560-6)
Supplement: Supplementary file 1 — Supplementary Information [file 41598_2021_81560_MOESM1_ESM.docx]

**Supplementary Information**

**Bioinspired antireflective flexible films with optimized mechanical resistance fabricated by roll to roll thermal nanoimprint**

Alejandra Jacobo-Martín^1^, Mario Rueda^2,3^, Jaime J. Hernández^1🖂^, Iván Navarro-Baena^1^, Miguel A. Monclús^2^, Jon M. Molina-Aldareguia^2🖂^ and Isabel Rodríguez^1^

^1^ Madrid Institute for Advanced Studies in Nanoscience (IMDEA Nanoscience), C/ Faraday 9, Ciudad Universitaria de Cantoblanco. 28049 Madrid, Spain

^2^ Madrid Institute for Advanced Studies in Materials (IMDEA Materials), C/ Eric Kandel 2, Tecnogetafe, Getafe. 28906 Madrid, Spain.

^3^ Department of Materials Science, Universidad Politécnica de Madrid, E. T. S. de Ingenieros de Caminos, 28040 Madrid, Spain

Figure S1 shows the AFM characterization of the moth-eye Ni reference mold. The mean measured nanocone height was estimated to be 335 nm.

| 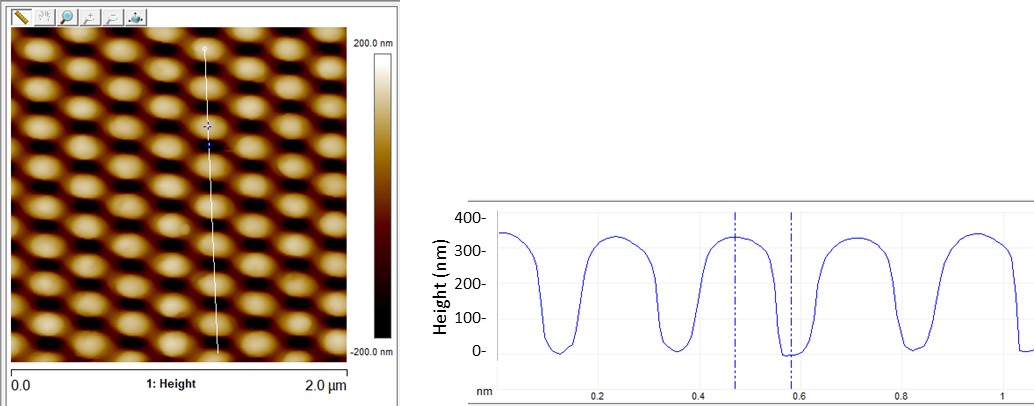 |
| --- |
| Figure S1. AFM characterization of the Ni mold with moth-eye like nanostructure. Topography 2D image (left) 1D height profile across the white line |

Figure S2 shows the set load profile and probe lateral displacement during scratch test.

| 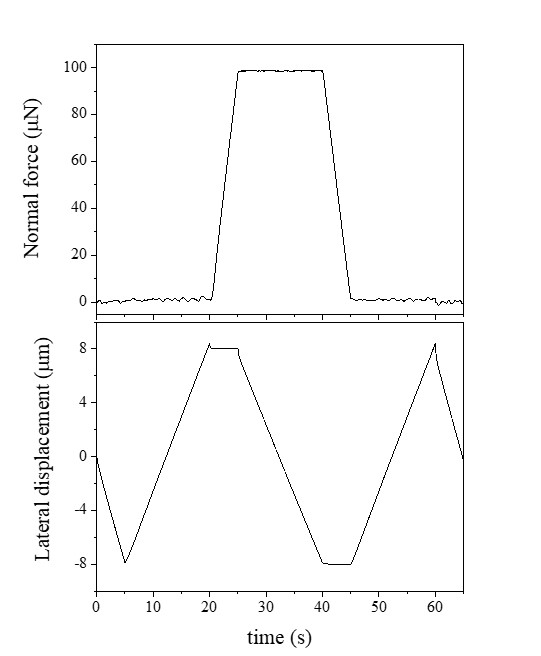 |
| --- |
| Figure S2. Set values for normal force (top) and probe lateral displacement (bottom) during nanoscratch measurement. |

Figure S3 shows the static water contact angle values of the films fabricated at 110ºC at different web speeds.

| 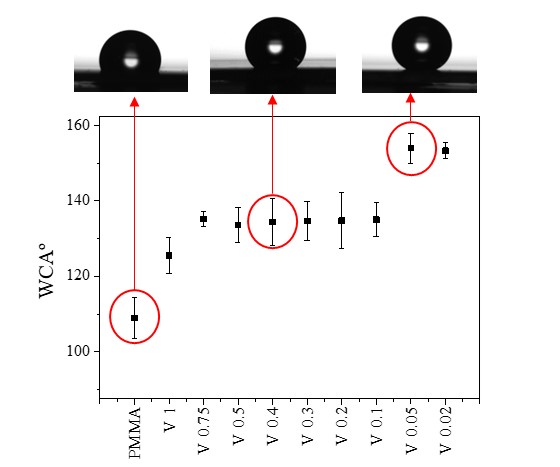 |
| --- |
| Figure S3. Static WCA values measured on nanostructured PMMA films prepared at varied web speeds and constant roll temperature of 110ºC. Selected pictures of water drops deposited over different substrates are shown. Drop size was set to 2.5 µl. |
